# A nomogram for predicting good response after neoadjuvant chemoradiotherapy for locally advanced rectal cancer: a retrospective, double-center, cohort study

**DOI:** 10.1007/s00384-022-04247-y

**Published:** 2022-09-01

**Authors:** Guancong Wang, Zhifang Zheng, Jinhua Chen, Jiahong Ye, Zihan Tang, Yicong Fang, Kaiyuan Yao, Qunzhang Zeng, Yugang Yang, Haiwen Tang, Bijuan Lin, Yincong Guo, Ying Huang

**Affiliations:** 1 Department of Colorectal and Anal Surgery, Zhangzhou Affiliated Hospital of Fujian Medical University, Zhangzhou, 363000 China; 2 Department of Colorectal Surgery, Fujian Medical University Union Hospital, Fuzhou, 350001 China; 3 Follow-Up Center, Fujian Medical University Union Hospital, Fuzhou, 350001 China

**Keywords:** Good response, Locally advanced rectal cancer, Neoadjuvant chemoradiotherapy, Nomogram

## Abstract

**Aim:**

The purpose of this study was to explore the clinical factors associated with achieving good response after neoadjuvant chemoradiotherapy (nCRT) in patients with locally advanced rectal cancer (LARC) and to develop and validate a nomogram.

**Methods:**

A total of 1724 consecutive LARC patients treated at Fujian Medical University Union Hospital from January 2010 to December 2021 were retrospectively evaluated as the training cohort; 267 consecutive LARC patients treated at Zhangzhou Affiliated Hospital of Fujian Medical University during the same period were evaluated as the external 2 cohorts. Based on the pathological results after radical surgery, treatment response was defined as follows: good response, stage ypT0∼2N0M0 and poor response, ypT3∼4N0M0 and/or N positive. Independent influencing factors were analyzed by logistic regression, a nomogram was developed and validated, and the model was evaluated using internal and external data cohorts for validation.

**Results:**

In the training cohort, 46.6% of patients achieved good response after nCRT combined with radical surgery. The rate of the retained anus was higher in the good response group (93.5% vs. 90.7%, *P* < 0.001). Cox regression analysis showed that the risk of overall survival and disease-free survival was significantly lower among good response patients than poor response patients, HR = 0.204 (95%CI: 0.146–0.287). Multivariate logistic regression analysis showed an independent association with 9 clinical factors, including histopathology, and a nomogram with an excellent predictive response was developed accordingly. The C-index of the predictive accuracy of the nomogram was 0.764 (95%CI: 0.742–0.786), the internal validation of the 200 bootstrap replication mean C-index was 0.764, and the external validation cohort showed an accuracy C-index of 0.789 (95%CI: 0.734–0.844), with good accuracy of the model.

**Conclusion:**

We identified factors associated with achieving good response in LARC after treatment with nCRT and developed a nomogram to contribute to clinical decision-making.

## Introduction

Rectal cancer with characteristics such as anatomical proximity to the sphincter structure, high local recurrence rate, and different metastatic behavior requires multiple means of combined and comprehensive treatment to obtain better outcomes [1]. In recent years, neoadjuvant chemoradiotherapy (nCRT) combined with total mesorectal excision (TME) has become the standard treatment mode for locally advanced rectal cancer (LARC) [2]. However, in clinical practice, the tumor response after nCRT in LARC patients varies according to individual differences, and some patients have a good tumor response; i.e., the tumor cells in radical surgical resection specimens infiltrate within the rectal muscular layer (ypT0∼2) and have no lymph node metastasis (N0) or even partially achieve a pathological complete response (pCR) [3]. This not only reduces the local recurrence rate but also results in a better prognosis. Meanwhile, selective ypT0∼2N0 patients are considered potential candidates for anal organ preservation [4]. However, due to the heterogeneity of rectal cancer, another proportion of patients respond poorly to nCRT, resulting in prolonged waiting time for surgery and increased risk of distant metastasis. Reportedly, 30∼40% of patients respond poorly after nCRT treatment, 20∼30% of patients fail to respond, and in a few cases, tumor progression even occurs [5].

Individually tailored treatment is urgently needed to develop tools that can accurately predict whether LARC patients will have a good response after nCRT treatment even before treatment decisions are made to help develop the best comprehensive treatment strategy in clinical work and allow some patients to be exempted from nCRT treatment [6]. The nomogram is a popular and well-visualized tool for predicting outcomes in the clinic and has been widely used to predict the different responses after nCRT among LARC patients [7, 8]. The purpose of this study was to explore the clinical factors associated with LARC patients achieving a good response after nCRT and to develop and validate a nomogram that can be used to predict good response before treatment decisions are made, as well as to assess the prognosis of good or poor tumor response.

## Patients and methods

### Patient selection

Consecutive patients with LARC treated at the Union Hospital of Fujian Medical University from January 2010 to December 2021 were retrospectively evaluated as the training cohort; consecutive patients with LARC treated at the Department of Colorectal and Anal Surgery of the Zhangzhou Hospital of Fujian Medical University during the same period were evaluated as the external validation cohort.

The inclusion criteria were as follows: ① pathologically confirmed rectal cancer; ② a clinical stage of cII or cIII (cT3∼4N0∼2M0) determined by two imaging experts according to the American Joint Committee on Cancer (AJCC) 8th edition colorectal cancer staging criteria; ③ completed nCRT combined with radical surgery; ④ complete clinicopathological features and follow-up data; and ⑤ signed informed consent form. The exclusion criteria were as follows: ① recurrence of rectal cancer; ② distant organ metastasis before treatment; ③ preoperative combined intestinal obstruction, perforation, intestinal bleeding, and other patients requiring emergency surgical resection; ④ death within 60 days after surgery; ⑤ first diagnosis combined with simultaneous or heterochronous malignant tumors from another organ. This study was approved by the Ethics Committee of Zhangzhou Affiliated Hospital of Fujian Medical University and Fujian Medical University Union Hospital (2020LWB078).

### Treatment

The comprehensive treatment plan was as follows: All patients received concurrent radiotherapy, with radiation therapy in the form of 3-dimensional conformal radiation therapy (3DCRT) or intensity-modulated radiation therapy (IMRT) and concurrent oral fluorouracil-based chemotherapy during radiotherapy. The long-course radiotherapy dose was 45.0∼50.4 Gy 25∼28 times, and the short-course radiotherapy dose was 25 Gy 5 times. The patients underwent neoadjuvant chemotherapy within 1 week following the completion of radiotherapy and while awaiting surgery. The neoadjuvant chemotherapy regimen was mFolFox6 (total fluorouracil 2600 mg/m^2^, calcium folinate 400 mg/m^2^, oxaliplatin 85 mg/m^2^) or Xelox (capecitabine 1000 mg/m^2^ bid, oxaliplatin 130 mg/m^2^). TME surgery was performed after a median time interval of 6–8 weeks after radiotherapy. Laparoscopic, robot, or open surgery was used.

### Definitions

The classification criteria were as follows: Specimens of all cases were reviewed by 2 pathologists in their units for pathological findings. Based on postoperative pathological examinations as criteria, treatment response was defined as follows: good response, stage ypT0∼2N0M0 and as the primary study endpoint in this study; and poor response, stage ypT3∼4N0M0 or N positive [3, 8]. pCR was defined as complete tumor regression, no found tumor cells, and those patients with only fibroblasts remaining would not receive postoperative adjuvant chemotherapy after surgery, whereas the remaining patients continued the total preoperative and postoperative courses of chemotherapy for a cumulative period of 6 months. After comprehensive treatment, follow-ups were conducted by telephone and outpatient visits. The follow-up period ended in June 2022.

### Statistical analysis

*T* tests, chi-square tests, and Fisher’s exact tests were used to analyze whether the differences between the training cohort and external validation cohort were statistically significant. Binary logistic regression analysis was used for variable analysis and selected variables as the basis for model construction. The Cox regression analysis method was used for the comparison of DFS and OS. SPSS 25.0 (IBM SPSS Statistics, Chicago, IL, USA) software was used for statistical analysis. *P* < 0.05 indicated that the difference was statistically significant. Independent factors were introduced into R 4.1.1 (http://www.r-project.org) software to create a nomogram using R packages such as car, pROC, and survivor. Internal validation of the model was performed using 200 bootstrap replications with put back sampling. External validation was also performed using validation cohort data. The accuracy of the model was judged by the consistency index (C-index). Calibration curves were used to evaluate the consistency of the model’s predicted results with the actual results. Decision curve analysis (DCA) was used to assess whether there was clinical benefit.

## Results

Finally, the training cohort included 1724 consecutive patients, and the external validation cohort included 267 consecutive patients. The general clinical characteristics of both groups are shown in Table 1. The two groups had good tumor responses of 46.6% and 43.8%, respectively, and the difference between the two groups was not statistically significant (*P* = 0.391); the follow-up times were 50.0 ± 24.2 months and 25.0 ± 22.79 months, respectively (Table 1).

**Table 1:** General clinical information of the training and external validation cohorts of patients with locally advanced rectal cancer (cases, %)

Variables	Training cohort (*n* = 1724)	Validation cohort (*n* = 267)	Total	*P*
Response				0.391
Poor	920 (53.4)	150 (56.2)	1070	
Good	804 (46.6)	117 (43.8)	921	
Sex				< 0.001
Female	845 (49.0)	100 (37.5)	945	
Male	879 (51.0)	167 (62.5)	1046	
Age				0.981
≤ 45 years	296 (17.2)	46 (17.2)	342	
> 45 years	1428 (82.8)	221 (82.8)	1649	
Histopathology				0.315
Signet ring cell carcinoma/mucinous adenocarcinoma	238 (13.8)	43 (16.1)	281	
Nonspecific adenocarcinoma	1486 (86.2)	224 (83.9)	1710	
Distance to the anal verge				0.030
≤ 8 cm	1510 (87.6)	221 (82.8)	1731	
> 8 cm	214 (12.4)	46 (17.2)	260	
Pre-CRT CA199				< 0.001
≤ 27 U/ml	1371 (79.5)	156 (58.4)	1527	
> 27 U/ml	353 (20.5)	111 (41.6)	464	
Pre-CRT CEA				0.120
≤ 5 ng/ml	1044 (60.6)	175 (65.5)	1219	
> 5 ng/ml	680 (39.4)	92 (34.5)	772	
Pre-CRT MRI CRM				0.003
Negative	670 (38.9)	129 (48.3)	799	
Positive	1054 (61.6)	138 (51.7)	1192	
Pre-CRT MRI EMVI				0.322
Negative	504 (29.2)	86 (32.2)	590	
Positive	1220 (70.8)	181 (67.8)	1401	
Pre-CRT MRI T stage				0.447
cT1	12 (0.7)	2 (0.7)	14	
cT2	124 (7.2)	14 (5.2)	138	
cT3	661 (38.3)	95 (35.6)	756	
cT4	927 (53.8)	156 (58.4)	1083	
Pre-CRT LN metastasis status				0.347
Negative	373 (21.6)	51 (19.1)	424	
Positive	1351 (78.4)	216 (80.9)	1567	
Combined molecular targeted neoadjuvant therapy				0.924
No	1636 (94.4)	253 (94.8)	1889	
Yes	88 (5.1)	14 (5.2)	102	
Total neoadjuvant therapy				< 0.001
No	1677 (97.3)	247 (92.5)	1924	
Yes	47 (2.7)	20 (7.5)	67	
Interval to surgery (weeks)	10.19 ± 3.40	10.57 ± 3.48		0.095^a^
Radiation therapy courses				0.864
Long-course	1616 (93.7)	251 (94.0)	1867	
Short-course	108 (6.3)	16 (6.0)	124	
Range of rectal wall circumference				0.517
Nonwhole	1404 (81.4)	213 (79.8)	1617	
Whole	320 (18.6)	54 (20.2)	374	

Abbreviations: *CRT* chemoradiotherapy, *CA199* carbohydrate antigen 199, *CEA* carcinoembryonic antigen, *MRI* magnetic resonance imaging, *CRM* circumferential resection margin, *EMVI* extramural venous invasion, *LN* lymph node

^a^*T* tests were used; the rest that are unlabeled used chi-square tests

In the training cohort, recent outcomes showed that while the tumors were lower relative to the anal verge in the patients with good response than in the patients with poor response (≤ 8 cm, 90.7% vs. 84.9%, *P* < 0.001), the patients who had a good response ultimately had a greater chance of preserving the anal sphincter (93.5% vs. 90.7%, *P* < 0.001). Among the patients who had a poor response, 9.6% suffered distant metastases after nCRT treatment (Table 2).

**Table 2:** Surgical treatment of patients with good response versus poor response in the training cohort (cases, %)

Variables	Poor response patients	Good response patients	*P*
Distance to the anal verge			< 0.001
≤ 8 cm	781 (84.9)	729 (90.7)	
> 8 cm	139 (15.1)	75 (9.3)	
Preservation of anal organs			0.028
No	86 (9.3)	52 (6.5)	
Yes	834 (90.7)	752 (93.5)	
Ostomy			0.132
Yes	355 (38.6)	282 (35.1)	
No	565 (61.4)	522 (64.9)	
ypTNM stage			< 0.001^a^
yp 0	0 (0.0)	386 (48.0)	
yp I	0 (0.0)	418 (52.0)	
yp II	436 (47.4)	0 (0.0)	
yp III	396 (43.0)	0 (0.0)	
yp IV	88 (9.6)	0 (0.0)	

^a^Fisher’s exact test

**Fig. 1:**
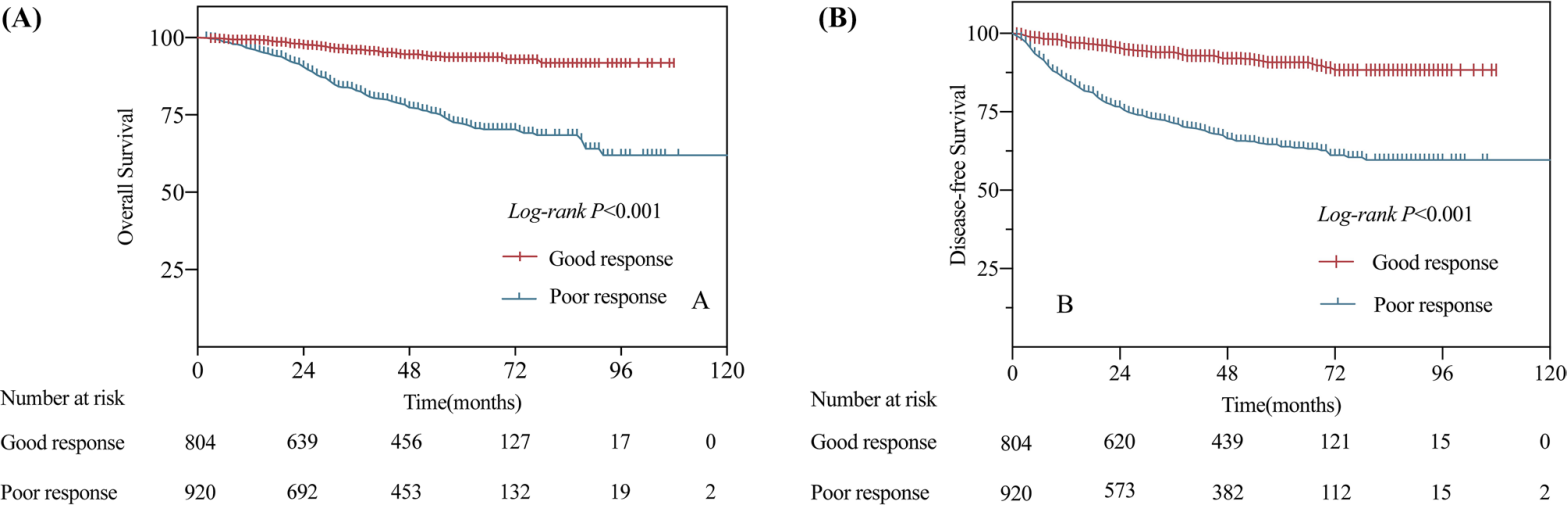
Survival curve plots of overall survival (**A**) and disease-free survival (**B**) for patients with good vs. poor response after nCRT for locally advanced rectal cancer in the training cohort

The long-term outcome was analyzed by survival curve results, and DFS and OS were significantly better among patients with good response than among patients with poor response (5-year OS: 93.7% vs. 72.1%; 5-year DFS: 90.8% vs. 63.8%; all *P* < 0.001), HR = 0.204 (95%CI: 0.146–0.287) (Fig. 1A, B).

Univariate logistic regression analysis indicated that the differences in histopathology, distance to the anal verge (DTAV), pre-CRT circumferential resection margin (CRM), pre-CRT magnetic resonance imaging (MRI), extramural venous invasion (EMVI), pre-CRT MRI T stage, pre-CRT lymph node metastasis status, total neoadjuvant therapy (TNT), interval to surgery, and whole circumference of the rectal wall were all statistically significant (all *P* < 0.05) (Table 3).

**Table 3:** Univariate and multivariate logistic regression analyses of factors associated with good response in the training cohort

	Univariate regression analysis		Multivariate regression analysis	
Variables	HR (95%CI)	*P*	HR (95%CI)	*P*
Sex (male vs. female)	1.133 (0.910–1.411)	0.264		
Age (> 45 years vs. ≤ 45 years)	1.104 (0.821–1.484)	0.513		
Histopathology (nonspecific adenocarcinoma vs. signet ring cell carcinoma/mucinous adenocarcinoma)	4.597 (3.122–6.769)	< 0.001	4.697 (3.216–6.862)	< 0.001
Distance to the anal verge (> 8 cm vs. ≤ 8 cm)	0.657 (0.471–0.916)	0.013	0.639 (0.460–0.889)	0.008
Pre-CRT CA199 (> 27 U/ml vs. ≤ 27 U/ml)	0.815 (0.614–1.082)	0.158		
Pre-CRT CEA (> 5 ng/ml vs. ≤ 5 ng/ml)	0.445 (0.353–0.560)	< 0.001	0.428 (0.343–0.535)	< 0.001
Pre-CRT MRI CRM (positive vs. negative)	1.036 (0.780–1.376)	0.809		
Pre-CRT MRI EMVI (positive vs. negative)	0.450 (0.338–0.599)	< 0.001	0.447 (0.338–0.591)	< 0.001
Pre-CRT MRI T stage (cT4, cT3, cT2 vs. cT1)	0.744 (0.595–0.929)	0.009	0.754 (0.621–0.914)	0.004
Pre-CRT LN metastasis status (positive vs. negative)	0.406 (0.310–0.531)	< 0.001	0.415 (0.317–0.542)	< 0.001
Combined molecular targeted neoadjuvant therapy (Yes vs. No)	0.717 (0.330–1.560)	0.402		
Total neoadjuvant therapy (Yes vs. No)	2.198 (1.177–4.105)	0.013	1.929 (1.121–3.318)	0.018
Interval to surgery (weeks)	1.050 (1.014–1.088)	0.007	1.055 (1.018–1.092)	0.003
Radiation therapy courses (short vs. long)	1.169 (0.755–1.811)	0.484		
Range of rectal wall circumference (whole vs. nonwhole)	0.442 (0.329–0.593)	< 0.001	0.446 (0.334–0.597)	< 0.001

Abbreviations: *CRT* chemoradiotherapy, *CA199* carbohydrate antigen 199, *CEA* carcinoembryonic antigen, *MRI* magnetic resonance imaging, *CRM* circumferential resection margin, *EMVI* extramural venous invasion, *LN* lymph node

Independent factors associated with good response were further analyzed. The results of multivariate logistic regression analysis showed that histopathological diagnosis of nonspecific adenocarcinoma (OR = 4.697, 95%CI: 3.216–6.862; *P* < 0.001), DTAV > 8 cm (OR = 0.639, 95%CI: 0.460–0.889; *P* = 0.008), pre-CRT CEA (carcinoembryonic antigen) > 5 ng/ml (OR = 0.428, 95%CI: 0.343–0.535; *P* < 0.001), positive pre-CRT MRI EMVI positive (OR = 0.447, 95%CI: 0.338–0.591; *P* < 0.001), pre-CRT MRI T stage cT4 (OR = 0.754, 95%CI: 0.621–0.914; *P* = 0.004), positive pre-CRT LN metastasis (OR = 0.415, 95%CI: 0.317–0.542; *P* < 0.001), total neoadjuvant therapy (OR = 1.929, 95%CI: 1.121–3.318; *P* = 0.018), interval to surgery (OR = 1.055, 95%CI: 1.018–1.092; *P* = 0.003), and whole circumference of the rectal wall (OR = 0.446, 95%CI: 0.334–0.597; *P* < 0.001) were independent influencing factors for good response (Table 3).

**Fig. 2:**
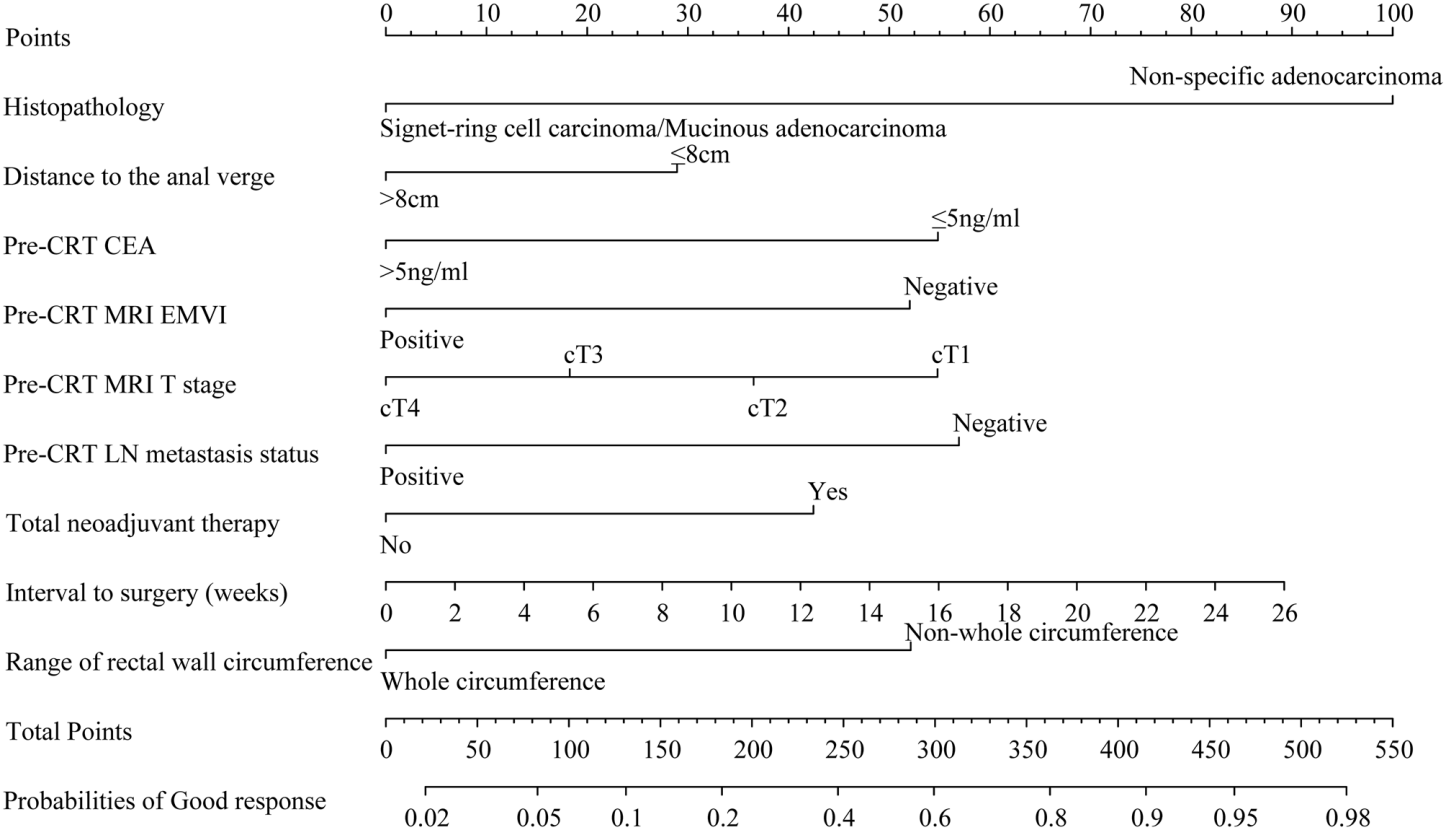
Nomogram for predicting good tumor response after neoadjuvant chemoradiotherapy for locally advanced rectal cancer

**Fig. 3:**
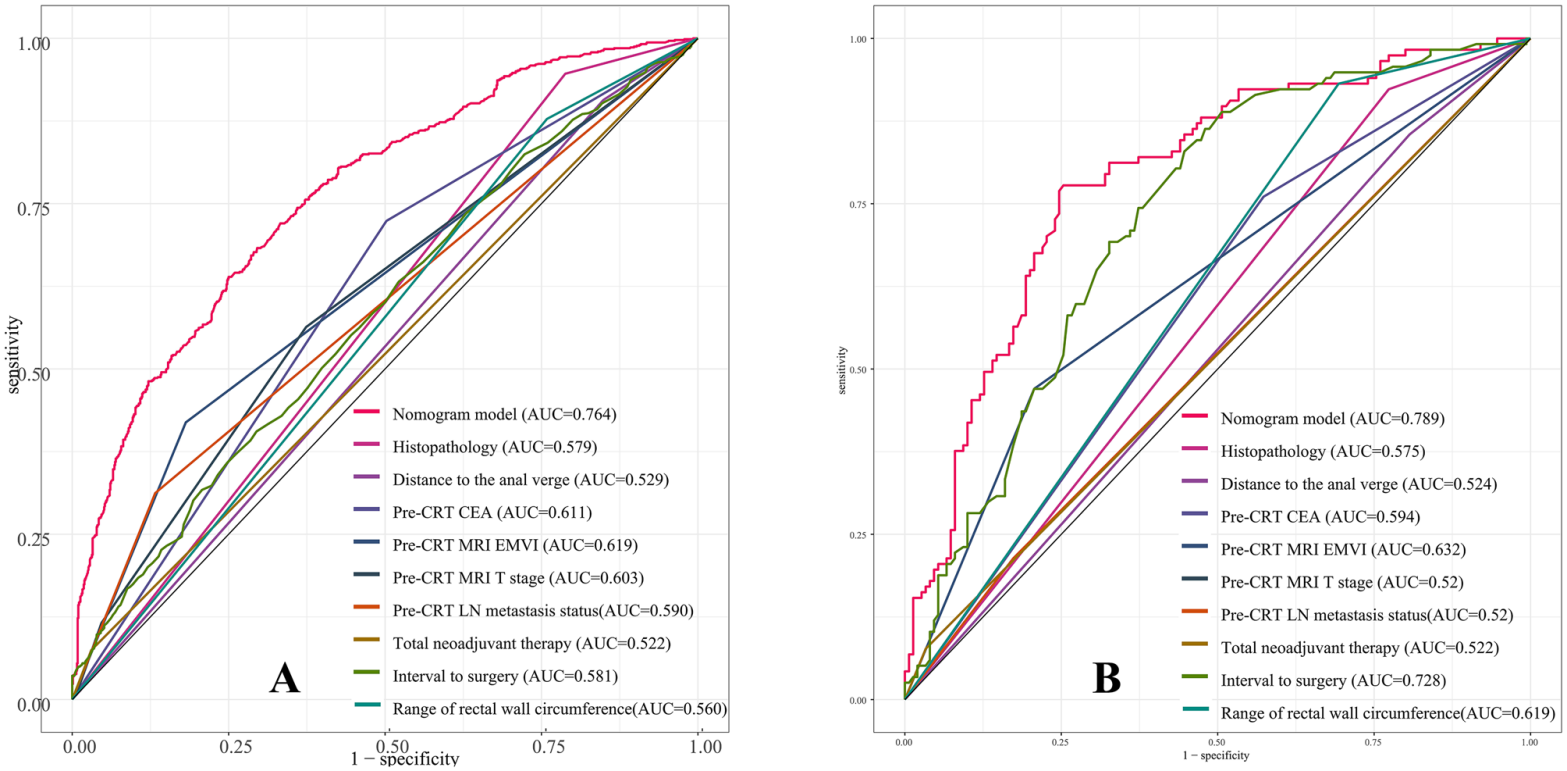
Area under the curve (AUC) for the training cohort (**A**) and external validation cohort (**B**) for good response

**Fig. 4:**
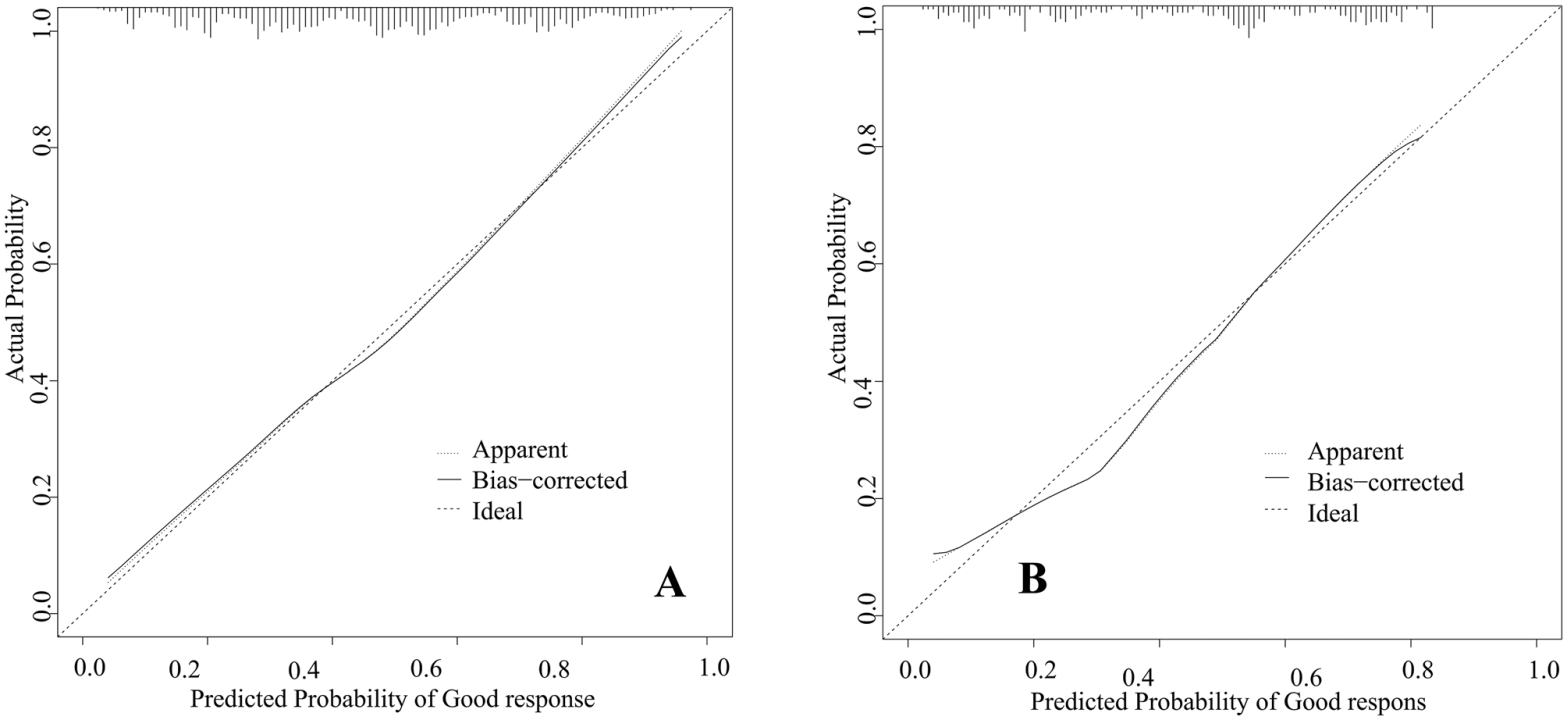
Calibration plots for the training cohort (**A**) and external validation cohort (**B**) for good response. The solid line represents the performance of the present nomogram, and the dashed line represents the performance of an ideal nomogram

**Fig. 5:**
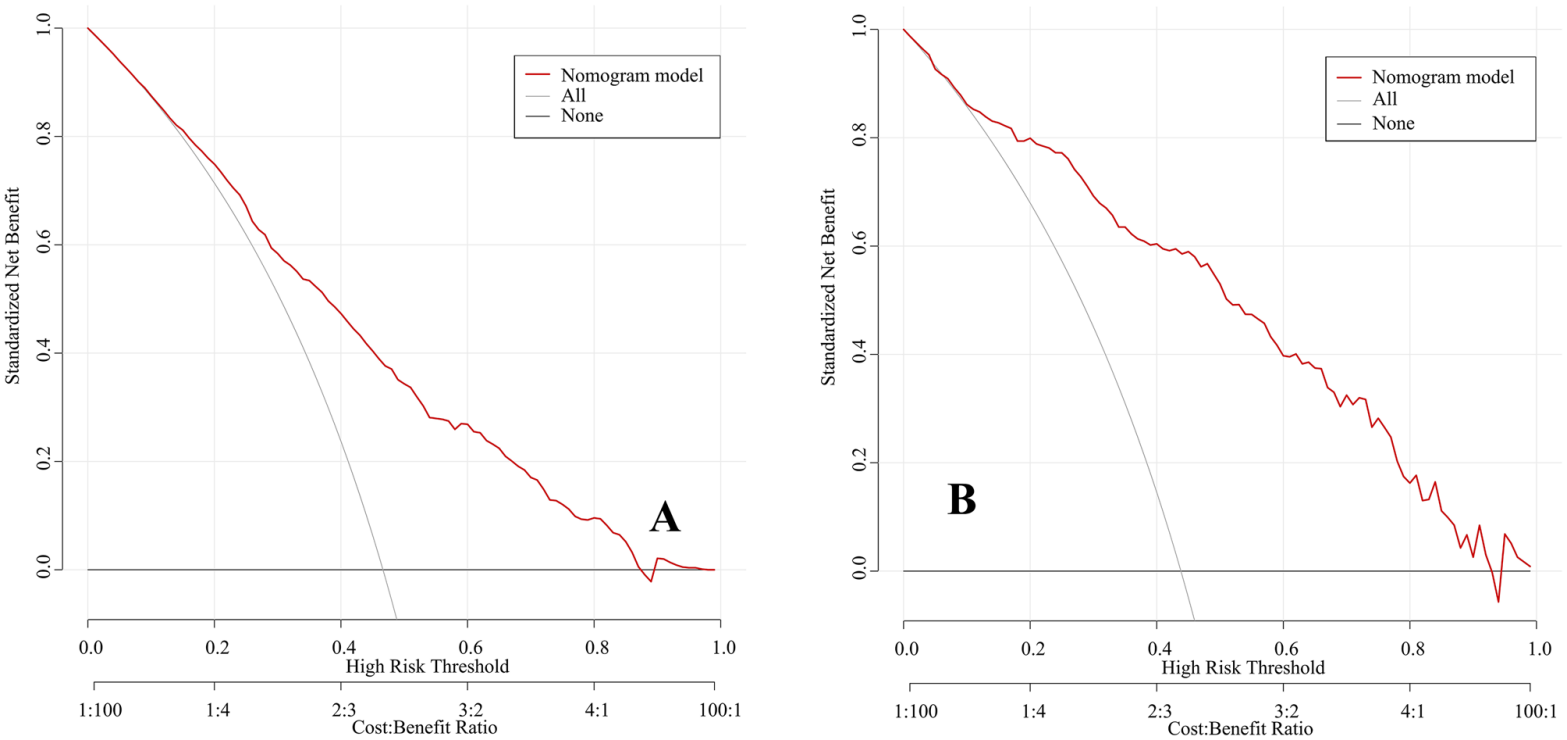
Decision curve analysis for the training cohort (**A**) and external validation cohort (**B**) for good response. All points are located above the None and All lines

Nine independent factors were included in the construction of the nomogram for LARC patients with good response after nCRT treatment (Fig. 2). The C-index of the predictive accuracy of the nomogram (Fig. 3A) was 0.764 (95%CI: 0.742–0.786), and the average C-index of the final model in internal validation using the 200 bootstrap replication sampling method was 0.764. The model had good accuracy. The external validation cohort (*n* = 267) showed an accuracy C-index (Fig. 3B) of 0.789 (95%CI: 0.734–0.844). The calibration curves (Fig. 4A, B) showed good agreement between the predicted probabilities and the actual observations of the obtained response prediction model for the training cohort and external validation cohort. The decision curve analysis (Fig. 5A, B) lies above both the None and All lines, quantitatively showing that the model has clinical utility.

## Discussion

Previous research mostly defined the study endpoint as pCR after LARC treated with nCRT [9, 10], but in practice, there are still difficulties in implementing methods including watch and wait and endoscopic transanalresection, even after achieving clinical complete remission (cCR); most patients still undergo TME surgery. Due to the excellent prognosis, patients defined as having a good response, including pCR and stage yp I patients, are the population with the greatest benefit after nCRT, but this population has rarely been studied, and no reliable predictive models are available. Therefore, this study retrospectively included consecutive patients from two major colorectal cancer consultation centers in China, and the study identified independent influencing factors for attaining good response after nCRT in LARC patients and established a nomogram to help clinical decision-making.

A meta-analysis that included more than 40,000 patients showed that the prognosis of patients after LARC treated with nCRT depended on the post-treatment pathological stage and was not related to the pretreatment clinical stage [11]. Tumor regression after nCRT can accurately predict whether patient prognoses can improve. The results of this study showed that 46.6% of patients in the training cohort had a good response, with significant downstaging or even pCR, and the long-term prognosis was significantly better than that in the poor response group, which is consistent with the results of previous studies [6]. Patients who achieve cCR or near cCR as assessed by strict selection criteria can be managed with watch and wait and endoscopic transanal resection to preserve the rectum [12]. In this study, compared to the patients with a poor response, a lower distance to the anal verge of the tumor was observed among patients with a good response (*P* > 0.05); however, more patients experienced preservation of the anal organ. The construction and application of this model has significant clinical significance. However, there is still no reasonable and effective screening tool to help select the right patients for the best treatment plan to improve treatment efficiency.

The development of a treatment plan for rectal cancer depends on the evaluation of high-resolution rectal MRI. Accurate clinical staging, including the depth of tumor infiltration, the presence of lymph node metastasis, CRM, and EMVI, is a prerequisite for deciding whether a patient should receive nCRT treatment. The efficacy of nCRT needs to be dynamically evaluated during the treatment process so that the treatment plan can be adjusted in time and the best timing of surgery can be achieved. Several studies have been devoted to exploring whether comprehensive pretreatment MRI features can be used for the early prediction of LARC response to nCRT, and studies have shown good predictive performance for models predicting treatment response, with AUC ranges as high as 0.793∼0.940 [13–15].

Wan et al. included 2267 LARC patients to compare the prognostic impact of different interval to surgery times, and as a result, fewer survival outcomes were observed in the shortest interval time group, and interval times longer than 13 weeks were associated with lower DFS rates (HR = 0.884, 95%CI: 0.778 to 0.921, *P* < 0.001) [16]. A meta-analysis evaluated the TNT regimen used for an extended interval time and showed that the pCR rate was 22.4% (95%CI: 19.4∼25.7%) and that TNT increased the odds of pCR by 40% (1.40, 95%CI: 1.08∼1.81, *p* = 0.010) [17]. As the application of nCRT expands and clinical research continues, it is becoming increasingly important to establish the optimal interval time between final radiotherapy and surgery to achieve the maximum number of patient benefits [18].

The impact of circumferential tumor location on neoadjuvant therapy has been rarely studied, and our center’s study was the first to find a higher proportion of pCR among patients with tumors located in the anterior rectal wall (26.7% in the anterior wall vs. 20.0% in the lateral wall vs. 12.3% in the posterior wall, *P* = 0.006), but the exact mechanism remains unknown [19]. Similar to the present study, it was concluded that patients with tumors occupying the whole range of rectal wall circumference had a lower proportion of good response, and tumors occupying different circumferential positions, including the lateral wall and posterior wall, in this group of patients with greater tumor burden may respond poorly to nCRT. This new finding helps to improve prediction accuracy and can be further studied in the future. Additionally, the shorter the DTAV was, the greater the use of neoadjuvant therapy (< 5 cm, 75.2% vs. 38.0%; *P* < 0.001) [20]; a DTAV ≤ 5 cm independently predicted the rate of tumor downstaging (OR = 2.66, 95%CI: 1.72–4.40, *P* < 0.001) [21]. Similar to this study, low rectal occupation was higher (≤ 8 cm, 87.6%), and there was an independent association between lower tumor location and good response (> 8 cm, OR = 0.639, 95%CI: 0.460–0.889, *P* = 0.008).

The nomogram, as a visual predictive tool, is easy to use and understand, and it can be easily used in the clinic to calculate the probability of disease occurrence and treatment efficacy and determine the prognosis of patients based on the graph [6, 7, 15, 22]. Some studies have compared nomograms with the traditional tumor-node-metastasis stage (TNM stage) system, which has specific advantages in determining treatment efficacy and tumor prognosis, even though tumors of the same stage still differ in terms of survival and local recurrence rates according to certain factors affecting prognosis [15, 22]. The model constructed in this study was internally and externally validated and confirmed to accurately and individually estimate the probability of whether patients will obtain good response after nCRT, which is a major advantage over the estimates provided by the current TNM system. The usefulness of this nomogram for clinical decision-making can be confirmed by decision curve analysis (DCA), which helps to guide the treatment choice more accurately for the benefit of the patient [23].

Based on very few previous studies of this population, most focused on predictions for pCR patients [3, 6, 7], Zhang et al. considered good response as a predictive endpoint and developed a nomogram model C-index of 0.760 (95%CI: 0.681–0.844), but the study had a smaller sample size, fewer included factors in the model, and no external validation, with lower predictive accuracy than the present study [8]. This study was based on a retrospective cohort analysis of patients from two large colorectal consultation centers to compensate for the predictive model for this population, and the model was applied in an external institution for validation, with some external applicability to help clinical decision-making. There are also limitations in this study, as it was based on a retrospective cohort analysis with some selection bias; patients with incomplete clinical data were excluded, and the probability of good response may be overestimated or underestimated. In the future, as research on colorectal cancer progresses, many new prognosis-related variables, including microsatellite status (MSS), RAS, and BRAF genes, will be gradually discovered. The nomogram model used to predict good response should also be updated to obtain more accurate predictive assessment efficacy.

In conclusion, LARC treated with nCRT had a high probability of a good response, and patients with a good response not only had a greater chance of preserving the anal organ but also had a better prognosis. The nomogram was created to help clinicians predict the likely outcome of treatment, reduce unnecessary radiation therapy for some patients, and help LARC patients choose the best treatment option.

## Data Availability

All data obtained or analyzed during this work are included within the article.
